# Methylsulfonylmethane (MSM) Supplementation in Adult Horses Supports Improved Skeletal Muscle Inflammatory Gene Expression Following Exercise

**DOI:** 10.3390/ani15020215

**Published:** 2025-01-14

**Authors:** Madison R. Barshick, Kristine M. Ely, Keely C. Mogge, Lara M. Chance, Sally E. Johnson

**Affiliations:** School of Animal Sciences, Virginia Polytechnic Institute and State University, Blacksburg, VA 24060, USA; kely1@vt.edu (K.M.E.); keelymogge@vt.edu (K.C.M.); lmc2563@vt.edu (L.M.C.)

**Keywords:** equine, skeletal muscle, methylsulfonylmethane, glutathione peroxidase, cytokine, transcriptome

## Abstract

Methylsulfonylmethane (MSM) is a sulfur-containing molecule with reported anti-inflammatory and antioxidant activities. Exercise causes the formation of free radicals and stimulates inflammatory gene expression in leukocytes and skeletal muscle. The hypothesis that dietary supplementation with MSM alters the exercise-mediated inflammatory and oxidant response was assessed in unfit adult thoroughbred geldings. Ten geldings (6.7 ± 1.6 yr) were assigned to a diet supplemented without (CON, *n* = 5) or with 21 g of MSM (*n* = 5) for 30 days. Following the supplementation period, horses performed a standardized exercise test (SET) with blood collections before (t = 0), 10 min, 1 h, 4 h, and 24 h post-SET. Skeletal muscle biopsies were retrieved from the middle gluteus before and 1 h post-SET for total RNA isolation. All horses were rested for 120 days before the experiment was repeated in a cross-over design. Plasma total antioxidant capacity was unaffected (*p* > 0.05) by either exercise or MSM. Plasma glutathione peroxidase activity was less (*p* < 0.05) in MSM horses than in the CON. Plasma IL6, IL8, IL10, and TNFα were unaffected (*p* > 0.05) by either exercise or diet. Transcriptomic analysis of skeletal muscle revealed 35 genes were differentially expressed (DEG; *p* < 0.05) by 2-fold or more in response to exercise; no MSM DEGs were noted. A comparison of the exercise by diet contrasts revealed that horses supplemented with MSM contained a greater number of exercise-responsive genes (630; logFC > 0.2; q < 0.05) by comparison to the CON (237), with many of these mapping to the immune response (71) and cytokine signal transduction (60) pathways. These results suggest supplementation of MSM as a dietary aid for improved anti-inflammatory responses in skeletal muscle following exercise.

## Simple Summary

Unfit horses performing exercise are especially at risk for oxidative damage and inflammation within skeletal muscle. Methylsulfonylmethane (MSM) is a commercially available dietary supplement that has been shown to reduce oxidative damage and inflammation caused by exercise in men. The hypothesis that MSM supplementation would function as an antioxidant and anti-inflammatory following exercise was tested in unfit adult horses. Horses were supplemented with or without MSM for 30 days, followed by a standardized exercise test (SET). Blood and skeletal muscle biopsies were collected to measure the markers of oxidation and inflammation. The results offer a conflicting story for MSM in regard to its antioxidant abilities. As an anti-inflammatory, MSM-supplemented horses had a greater number of exercise-responsive genes by comparison to the controls, indicating a role for MSM in the inflammatory process. Overall, the results suggest MSM supplementation for improved anti-inflammatory responses in skeletal muscle following exercise.

## 1. Introduction

Skeletal muscle damage can occur at various degrees in response to physical exercise. Hallmarks of damage include sarcomere streaming, perturbation of the sarcolemma, and release of small myogenic molecules into the bloodstream [1,2]. Skeletal muscle repair occurs in a sequential manner, starting with an acute inflammatory response, denoted by neutrophil and monocyte invasion, following the conclusion of the exercise bout [3]. Infiltration of leukocytes and macrophages (M1) contributes to the increased expression of pro-inflammatory cytokines, including IL1β, IL6, IL8, and TNFα, in the muscle. Increasing concentrations of these cytokines serve as a feed-forward mechanism to attract additional immune cells to debride the region and clear damaged tissue. The polarized M2 macrophage replaces the M1 population following the clearance of damage and signals the synthesis and secretion of anti-inflammatory cytokines within the fiber niche. The presence of anti-inflammatory cytokines (IL4, IL10, G-CSF) within the niche creates a permissive environment for the muscle stem and progenitor population (satellite cells; SCs) that facilitates their bioactivities and permits fiber repair [4]. Although the process is firmly established in response to necrotic death and severe injury, the interplay between immune function and post-exercise skeletal muscle repair is less understood [5].

Facilitation of proper crossbridge cycling during intense exercise requires substantial ATP generation by mitochondrial oxidative phosphorylation. Both increased mitochondrial respiration and sarcomere shortening cause the formation of reactive oxygen species (ROS), a class of molecules that includes both free radicals and non-free radicals [6]. The formation of ROS causes lipid membrane peroxidation and oxidation of proteins that disrupt normal muscle contraction and metabolism. Muscles experience reductions in power output and fatigue as an outcome of disruptions to normal function [7]. Countermeasures to a perturbed redox status include the dietary supplementation of antioxidants. Conjugated linoleic acid, amino acid conjugates of copper, zinc, manganese, vitamin E, and selenium offer protective advantages to horses experiencing oxidative stress by reducing lipid peroxidation in muscles and other tissues [8,9,10]. Athletic horses, like their human counterparts, may benefit from the use of antioxidant and anti-inflammatory aids during the post-exercise recovery period.

Methylsulfonylmethane (MSM) is a naturally occurring compound that is commonly administered in the diet as an antioxidant and anti-inflammatory [11]. Compared to consumption of a placebo, consumption of MSM resulted in lower lipid peroxidation byproducts following exercise in men, supporting the role of MSM as an antioxidant [12]. Other research reports an increase in systemic IL10, an anti-inflammatory cytokine, following a bout of exercise in men receiving MSM supplementation, supporting the role of MSM in response to inflammation [13]. Additionally, MSM may act analogous to non-steroidal anti-inflammatory drugs by suppressing pain. Both sedentary healthy men and women reporting mild knee pain [14] and men completing a single bout of exercise [15,16] reported less soreness in the afflicted tissue after consuming MSM for at least 28 days. Fit jump horses consuming MSM for as little as 7 days demonstrated a retention of plasma redox capacity following exercise by comparison to non-supplemented horses [17]. Research regarding the effects of MSM on inflammation in equine athletes following exercise remains unsubstantiated.

Deficiencies in our understanding of the effects of MSM on systemic inflammatory cytokine profiles, antioxidant capacity, and skeletal muscle gene expression following exercise exist in horses. To fill these gaps in knowledge, MSM was supplemented to adult thoroughbreds, and their inflammatory responses and antioxidant capacity were evaluated in the post-exercise period. It was hypothesized that MSM supplementation to adult thoroughbreds for 30 days would decrease the pro-inflammatory response and increase the antioxidant capacity in the post-exercise period.

## 2. Materials and Methods

All animal procedures were reviewed and approved by the Virginia Tech Institutional Animal Care and Use Committee (23-220).

### 2.1. Experimental Treatments and Exercise

Ten adult, clinically sound, and previously inactive thoroughbred geldings (6.7 ± 1.6 yr; 526 ± 38 kg) were separated into groups of 3 and maintained on a 24 h turnout in 0.2 ha dry lot paddocks for the course of the study. Horses within pens were randomly assigned to a diet with or without 21 g of methylsulfonylmethane (MSM; Mad Barn, Kitchener, ON, Canada) as a top-dress per the manufacturer’s recommendation. Horses were fed orchard grass hay at 1.8% of BW and a commercial grain (Ultium Competition, Purina, Gray Summit, MO, USA) at 0.2% of BW daily. Each horse received the grain diet individually to ensure the consumption of MSM. All horses had ad libitum access to a mineral supplement (Purina Free Balance 12:12 Vitamin & Mineral Supplement, Purina, Gray Summit, MO, USA). The grain volume was adjusted as needed to maintain a constant BW and a target body condition score of 5.0 on a 9.0 scale [18]. The nutrient composition of the provided diet is shown in Table 1 and was designed to meet the maintenance requirements of adult thoroughbred horses (NRC, 2007). Following a 3-day sedentary supplementation period, horses performed a standardized exercise test (SET) on a high-speed treadmill (EquiGym, Paris, KY, USA). The SET consisted of 2 min at 2 m/s, 4 min at 4 m/s, 6 min at 10 m/s and 5 min at 4 m/s. Upon completion of the experiment, the horses were maintained on stockpiled fescue pastures with ad libitum access to water, trace mineral salt (Purina Free Balance 12:12), and grain (Table 1) supplementation as needed to maintain a minimum BCS of 5.0 for 120 days before repeating the above in a cross-over experimental design.

### 2.2. Sample Collection

Blood (8 mL) was collected into heparin- and EDTA-containing tubes (BD Vacutainer, Thermofisher, Waltham, MA, USA) before (t = 0), 10 min, 1 h, 4 h, and 24 h after the SET. Plasma was isolated from whole blood following centrifugation at 1500× *g* for 15 min, aliquoted, and stored at −80 °C. Muscle biopsies were retrieved from alternate sides of the middle gluteus muscle 12 h prior to and 1 h after completion of the SET [19,20]. Horses were sedated with xylazine (Bimeda-MTC Animal Health, Cambridge, ON, Canada), and the skin atop the muscle was shaved and surgically prepared with chlorohexidine (Nolvasan Scrub, Zoetis, Kalamazoo, MI, USA). The location of the biopsy site was identified by tracing an imaginary line between the tubercoxae and sacrococcygeal joint with sample retrieval between the first third and the center point of the line [20]. The biopsy region was anesthetized by subcutaneous injection of 2% lidocaine (VetOne, Biose, ID, USA), and a 1 cm incision through the skin was created with a sterile scalpel. A 12-gauge, vacuum-assisted biopsy device (Mammotome, Cincinnati, OH, USA) was inserted through the incision, and approximately 300 mg of tissue was retrieved. The muscle was briefly rinsed free of blood with sterile phosphate-buffered saline (PBS) and flash-frozen in liquid nitrogen. The muscle samples were stored at −80 °C until use.

### 2.3. Cytokine Assays

A previous project within the lab indicated that most cytokine concentrations within the plasma samples fell outside the linear range of the assay. Thus, 0.5 mL of plasma was concentrated by ultrafiltration through spin columns with a molecular weight cutoff of 3 kDa (Pierce™ Protein Concentrators PES, Thermofisher, Waltham, MA, USA) to ensure that the cytokine concentrations fell within the linear range of the assay. Cytokines were quantified in the retentate using a Luminex bead array assay (EQCYTMAG-93K; Millipore, Burlington, MA, USA), performed according to the manufacturer’s instructions and read on an Intelliflex DR-SE instrument (Luminex; Thermofisher). Data were analyzed using Bio-Plex manager v6.2 (Bio-Rad, Hercules, CA, USA).

### 2.4. Biochemical Assays

Blood lactate from the plasma samples obtained before (t = 0), 10 min, 1 h, and 4 h post-SET was analyzed using a colorimetric assay (MAK329, Sigma Aldrich, St. Louis, MO, USA), with the measurements obtained at an absorbance reading of 570 nm. The total antioxidant capacity (TAC) from the plasma samples obtained before (t = 0), 1 h, and 4 h post-SET was analyzed using a colorimetric assay (STA-360, Cell Biolabs, Inc., San Diego, CA, USA), with absorbance measures at 490 nm. Glutathione peroxidase (GPx) activity from the plasma samples obtained before (t = 0), 1 h, and 4 h post-SET was analyzed using a colorimetric assay (703102, Cayman Chemicals, Ann Arbor, MI, USA) at an absorbance reading of 340 nm.

### 2.5. RNA Isolation and Transcriptome Analysis

Total RNA was isolated using TRIzol (Thermofisher) coupled with spin-column purification (PureLink RNA, Thermofisher), as described previously [19]. Total RNA (RIN > 8.0) was used for polyA mRNA isolation and cDNA library construction. Adapters and low-quality base reads were removed from the raw sequencing reads using fastp (v0.23.1). The trimmed reads were aligned to the reference genome (GCF_002863925.1) using the STAR aligner (v2.7.11). Quantification was performed using Salmon (v1.10.2) at the transcript level. The R tximport package (v1.30.0) was utilized to import the transcript-level quantification results and to summarize the reads at the gene level. Statistical computation was performed using the Omics Playground (v 3.5.0). Gene-level testing was completed within the Omics Playground using multi-method statistical testing for the main effects of diet and exercise and exercise X diet contrasts [21]. Statistical significance was tested using the following three independent statistical methods: DESeq2 (Wald test), edgeR (QLF test), and limma-trend. The false discovery rate was determined as the aggregate q-value between the three statistical methods and correlates to the intersection of significant genes between all three tests. The Benjamini–Hochberg corrected false discovery rate of q < 0.05 was used for all bioinformatic analyses.

Pathway analysis was performed in REACTOME (v90) with default human gene identifiers using DEGs from the gene-level testing [22,23]. Pre-analysis first mapped the DEGs submitted as HGNC gene symbols to Reactome molecules and pathways within the database. Over-representation and pathway-topology analyses were then performed. Hypergeometric distribution determined the over-represented Reactome pathways and assigned each result a probability score. The probability scores were corrected for false discovery rates using the Benjamini–Hochberg method. Pathway-topology analysis was used to consider molecule connectivity within the Reactome pathways.

### 2.6. Statistical Analysis

Data were analyzed as a two-way ANOVA with repeated measures for the effects of treatment, exercise, and the treatment X exercise interaction. Multiple comparison analysis was performed using Sidak’s multiple comparison test with a pooled variance. Significance was established at *p* ≤ 0.05 for all analyses, with tendencies established at *p* > 0.05 ≤ 0.1.

## 3. Results

**Figure 1:**
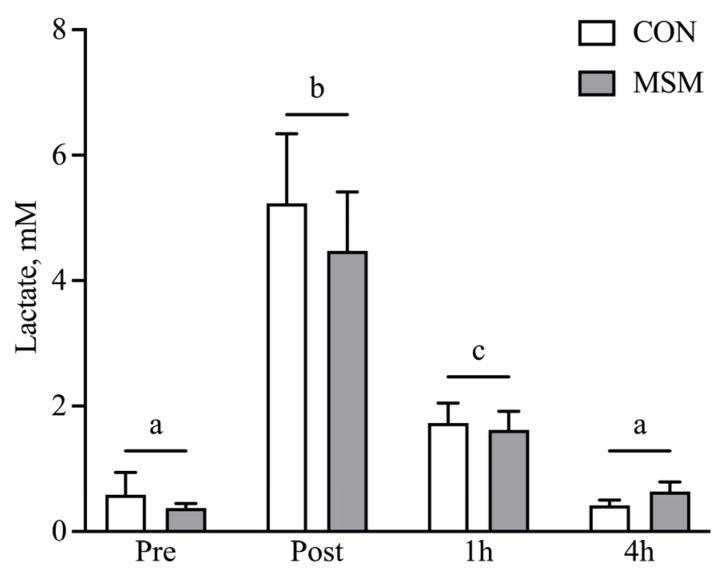
Blood lactate response following a submaximal SET test. Blood lactate increases at 10 min post-exercise, followed by a decline to baseline by 4 h following a submaximal SET test. MSM treatment did not affect blood lactate prior to or following exercise. Means and SEMs are shown. Different letters indicate significance at *p* < 0.05.

The extent of the exercise stress was evaluated by lactate production post-exercise. The test was designed to mimic the conditions of a lower-level event competition with plasma lactate levels ranging from 2.5 to 7 mM [24,25]. Although not directly measured, the maximum heart rate for sedentary horses performing a similar test is 192 ± 20 bpm (unpublished data). Diet did not impact lactate production at any time before or after the SET (Figure 1). Blood lactate concentrations were elevated (*p* < 0.05) at 10 min post-exercise and returned to baseline by 4 h post-SET.

**Figure 2:**
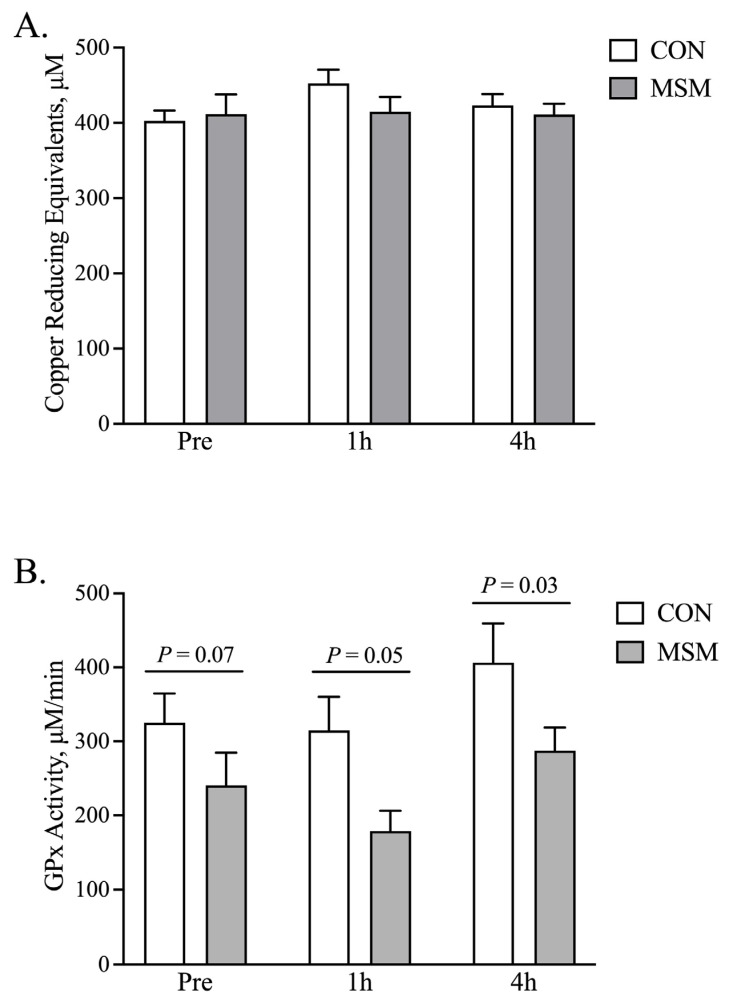
MSM supplementation modified GPx activity without changing total antioxidant capacity following a single SET. Following a single SET, dietary supplementation with MSM did not significantly affect the total antioxidant capacity within plasma (**A**). Glutathione peroxidase activity was reduced by MSM supplementation (*p* < 0.05) and tended to be less with time post-SET (**B**). No diet-by-time interactions were noted.

Plasma isolated from the MSM and CON horses was examined for total antioxidant capacity (TAC). Neither time nor dietary MSM altered TAC’s activity (Figure 2A). By contrast, both diet (*p* < 0.05) and time (*p* < 0.08) altered glutathione peroxidase’s (GPx) activity; no diet-by-time interaction was noted. The addition of MSM to the diet caused a reduction (*p* < 0.05) in glutathione peroxidase (GPx) activity by comparison to the CON at 1 h and 4 h post-SET with a tendency for lower activity (*p* < 0.07) prior to exercise (Figure 2B).

**Figure 3:**
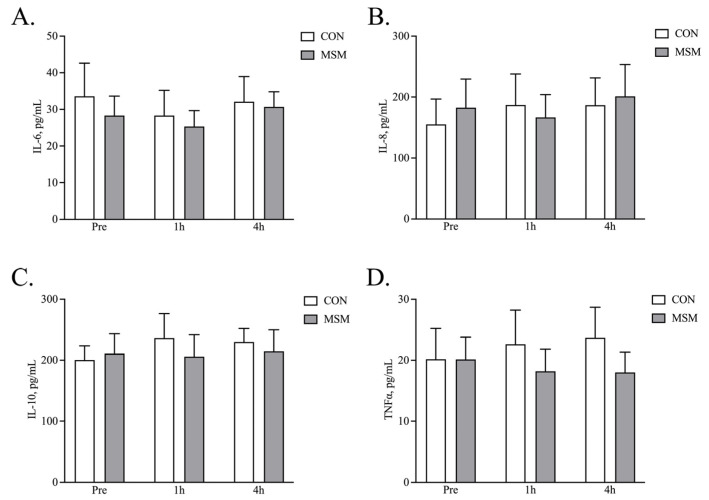
Plasma cytokine concentrations post-SET. Plasma samples were concentrated prior to being analyzed for cytokine concentrations using a bead-based immunoassay. Neither treatment (MSM supplementation) nor exercise altered IL6 (**A**), IL8 (**B**), IL10 (**C**), or TNFα (**D**) concentrations.

The ability of MSM to function as an anti-inflammatory was assessed by measurement of the plasma cytokine concentrations before (t = 0), 1 h, and 4 h after the SET. Bead-based immunoassays revealed neither treatment (MSM) nor time post-exercise altered the concentrations of IL6, IL8, IL10, or TNFα (Figure 3A–D).

**Figure 4:**
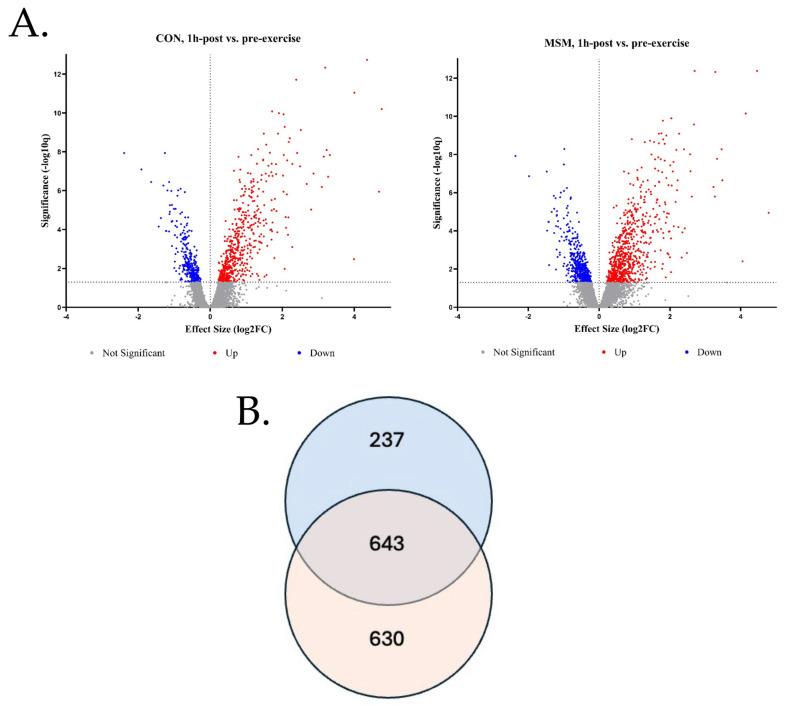
Comparison of the pre- vs. post-exercise contrast for the diets revealed divergent expression between MSM- and CON-supplemented horses. Volcano plots of the pre- vs. post-exercise contrasts for the diets show a greater number of blue (downregulated) and red (upregulated) dots for the right MSM-supplemented plot compared to the left CON-supplemented plot (**A**). Further comparison of these contrasts revealed 630 genes (red, bottom) with different response levels in MSM-supplemented horses by comparison to 237 genes (blue, top) in CON horses (**B**). Both treatments shared 643 common DEGs (overlap). FDR was set to q = 0.05 and genes were included with a logFC > 0.2.

**Figure 5:**
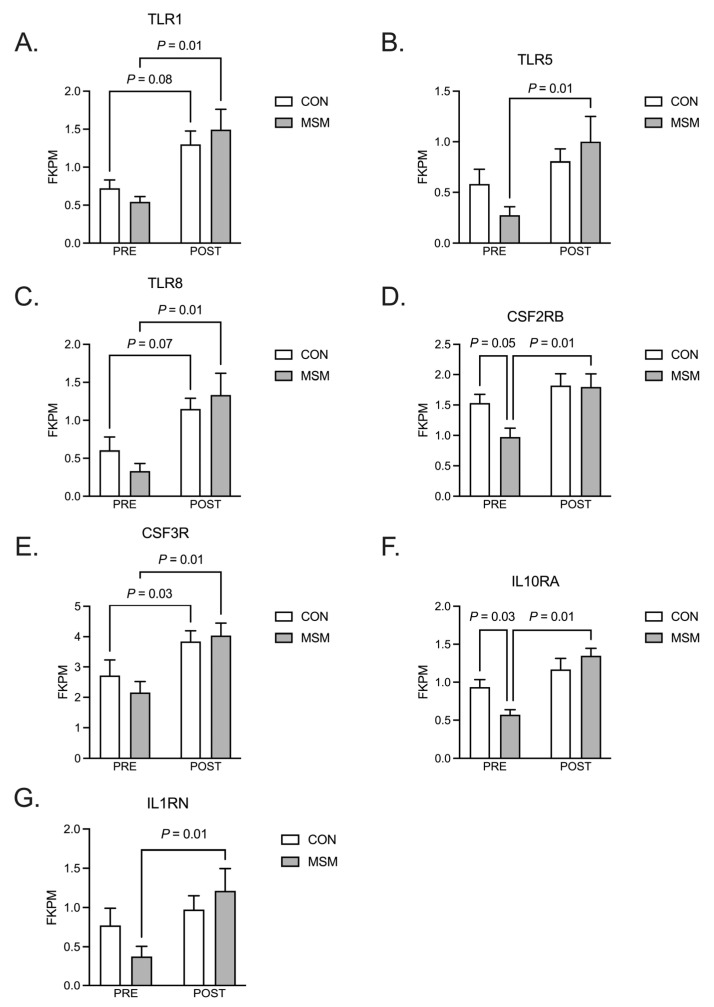
Select receptor genes involved in Toll-like receptor, interferon, and interleukin signaling pathways, which were modified with MSM supplementation. Reactome pathway query indicated that within DEGS from MSM-supplemented horses, there was an over-representation of immune response and cytokine signaling pathways. Select receptor genes from these specific pathways were selected for visualization. TLR1 (**A**), TLR5 (**B**), TLR8 (**C**), CSF2RB (**D**), CSF3R (**E**), IL10RA (**F**), and IL1RN (**G**) were all upregulated following exercise in MSM-supplemented horses. CSF2RB and IL10RA expression was modified between MSM and CON-supplemented horses prior to exercise (**D**,**F**).

Transcriptome analysis was performed to examine the antioxidant and anti-inflammatory gene expressions in skeletal muscle as a function of exercise. The treatment (MSM) did not elicit an effect on gene expression (q = 0.05; *p* > 0.10). As expected, exercise provoked gene expression in the skeletal muscle. One hundred ninety-four (194) genes were upregulated by the exercise stressor, and the expression of 26 genes was reduced (q = 0.05, logFC ≥ 1). Genes exhibiting the greatest change (logFC ≥ 2) are reported in Table 2. A visual comparison of the volcano plots (Figure 4A) for the CON and MSM demonstrates similar patterns for up- and downregulated genes, which were slightly more evident in the MSM group. To gain insight into the relative change in exercise response genes as a function of diet, the pre- vs. post-exercise contrast for both diets was compared. The analysis revealed 630 (q = 0.05, logFC ≥ 0.2) genes demonstrated divergent expression in MSM-supplemented horses by comparison to 237 genes in the CON horses, offering a distinct expression pattern between groups (Figure 4B). Common exercise responses were observed for 643 genes. The Reactome database (www.reactome.org accessed on 10 September 2024) was queried with the MSM response genes to identify the pathways affected by the molecule as a function of exercise. Of the 630 genes queried, 392 were mapped to a known pathway within the database. The pathway containing the greatest number of mapped genes (132) was *Immune System* (Table 3). Hierarchal clusters of genes demonstrated further over-representation in Toll-like receptor cascades (15), interferon signaling (11), and interleukin signaling (24) pathways. Select receptors from these subgroups were examined for expression abundance (Figure 5). In all instances, each receptor mRNA was upregulated (*p* = 0.01) post-exercise in the MSM-supplemented horses (Figure 5A–G). Expression of CSF2RB, CSF3R, and IL10RA also increased (*p* < 0.05) in response to exercise (Figure 5D–F). Toll-like receptors 1 and 8 expression tended (*p* = 0.07) toward upregulation in the CON following exercise (Figure 5A,C). A similar database query was performed using the 237 genes within the CON group that responded differently to the SET. A total of 134 genes were mapped to pathways within the Reactome database, with genes involved in the general identifier classes of *Metabolism* (30) and *Signal transduction* (42) being the most over-represented (Table 4). Due to the limited enrichment within these groups and subgroups, further analyses were not pursued.

## 4. Discussion

Reactive oxygen species formation is a common and necessary response to exercise. Although ROS production can lead to membrane damage, the class of molecules also serves as intracellular signaling intermediates that initiate and support the post-exercise muscle repair process [26]. Correct maintenance of the redox status becomes critical to offset ROS damage while allowing reparative activities. Blood GPx activity is often used as a proxy for redox status in response to exercise, stress, and/or diet, though GPx activity can be modified by fitness. Enzymatic activity of GPx is reduced by approximately 40% in unfit adult horses immediately following a single bout of submaximal exercise with a return to baseline within an hour [27]. By contrast, horses trained to an equivalent fitness level experienced no change in plasma GPx activity as a function of exercise [28,29]. Our results offer an ambiguous picture with only a tendency for reduced GPx activity at the 1 h timepoint that returns to normal by 4 h in unfit horses. Without a clear picture of the post-exercise serum GPx, it may be difficult to establish the influence of MSM on redox activity. The literature suggests that MSM does provide some level of antioxidant activity. Indeed, an increase in serum protein carbonyls and lipid peroxides occurs in unfit men within 30 min of exercise that is prevented by ingestion of MSM for 10 days [12]. Fit show jumpers exhibit reduced plasma GPx activity following a single bout of exercise that is counteracted by MSM supplementation [17]. By contrast, the serum total antioxidant capacity 2 h after exercise did not differ between men receiving either a placebo or MSM [15]. Our results exhibit no effect of MSM on the systemic measures of redox capacity (TAC, GPx), suggesting that the supplement offers little benefit in unfit horses. The fitness level, species, age, and exercise intensity are variables that need to be considered, however, before drawing firm conclusions on the value of MSM as an antioxidant.

The inflammatory response to acute exercise in men and mice is unequivocal; an increase in leukocyte and neutrophil accumulation in the muscle, coupled with increased cytokine levels (TNFα, IL1β, IL6, IL8, and INFƔ) signifies an initial pro-inflammatory state. The transition to an anti-inflammatory environment is denoted by macrophage polarization and the secretion of IL10 [30]. Altered muscle cytokine expression post-exercise, however, is not reflected faithfully in the blood cytokine profiles. Serum levels of pro-inflammatory cytokines (IL6, IL8) are greater [31,32] or remain unchanged [33] after intense exercise in clinically healthy active men and women, with all reporting no changes in their systemic TNFα concentrations. Horses offer mixed results regarding the blood cytokine profiles after exercise, as well. Fit immature horses (12–18 mos.) exhibited increased IL1β, IL6, IL8, IL10, and TNFα within an hour of completing an exercise stressor [34]. Others reported no change in their serum pro-inflammatory cytokines (TNF, IL6, and IL1β) as a function of short and intense exercise [35], moderate-intensity work [36], or simulated race conditions [37]. Our results fall within the scope of the later studies, with no discernable changes in the plasma IL6, IL8, or TNFα concentrations. Caution should be noted, however, as measurements of blood cytokines demonstrate high inter-horse variations, with evidence of interfering substances present within both the serum and plasma [38]. Values for resting baseline pro-inflammatory cytokines (IL1β, IL4, IL6, and TNFα) range from 100 pg/mL to 10 ng/mL, with some horses exhibiting 10–100-fold greater concentrations and others falling below the detection limits. The safest approach to assessing the systemic inflammatory response to exercise likely remains the measurement of leukocyte cytokine mRNA profiles, as suggested over 20 years ago [39,40].

A clear exercise effect was observed in the skeletal muscle, with many of the upregulated genes being part of a global exercise response reported in multiple species. In the horse, expression of PDK4 [41,42] and the heat shock protein family of genes (HSPA1, HSPH1, DNAJB1) are upregulated in response to exercise [43,44]. Nuclear hormone receptor 1 subfamily 4 group A3 (NR4A3) was the maximally expressed gene in response to exercise (4.9-fold), with its paralog family member NR4A2 exhibiting a similar increase in expression (4.4-fold). The NR4A subgroup of orphan nuclear receptors lacks an identifiable ligand but is rapidly transcribed in immune cells following mitogen stimulation [45]. In human macrophages, the transcription factor NR4A3 stimulates gene expression typical of anti-inflammatory M2 cells, including IL1RA and IL10 [46]. Moreover, an extensive meta-analysis of human muscle transcriptome profiles (>50 data sets) identified NR4A3 as a nodal point and primary driver of the acute exercise response [47]. Ectopic expression of NR4A3, specifically within the muscle fiber, leads to mitochondria biogenesis and increased numbers of type I and IIA oxidative fiber in mice [48], while myocytes in vitro contain reduced amounts of myosin I and IIA following the knockdown of NR4A3 mRNA [49]. Our results extend those from human and mouse studies to include the horse and suggest that NR4A3 may work to promote an anti-inflammatory state within the fiber niche while facilitating muscle mitochondria function during the post-exercise period.

Methylsulfonylmethane supplementation did not modify basal skeletal muscle gene expression but did affect the degree of transcriptional responses for several genes involved in the innate immune response. Increased transcription of the genes associated with TLR, interferon, and interleukin signaling was noted post-exercise, with MSM supplementation augmenting their expression. The overall effect of exercise runs counter to those found in men and women, where no changes in TLR1, 5, or 8 are evident following an acute bout of resistance exercise [50,51]. However, others report that healthy men and women consuming an antioxidant supplement containing MSM expressed greater amounts of several pro-inflammatory genes, including TLR5, in leukocytes following aerobic exercise (marathon) [52]. Stimulation of primary human myocytes with a TLR5 ligand caused an increase in IL6, IL8, and GM-CSF transcription and secretion in vitro [53]. An attractive hypothesis is that MSM enhances the TLR5-stimulated production of pro-inflammatory cytokines that hastens exercise-induced damage repair. Shifting the timeframe of repair can lead to macrophage polarization (M2) and secretion of anti-inflammatory cytokines (IL10 and IL1RA) and growth factors (TGFβ and IGF1) [54,55,56]. In a septic mouse model, MSM promoted M2 formation and increased IL10 secretion [57]. Greater expression of CSF3R in horses receiving MSM may indicate increased muscle stem cell (satellite cells, SCs) activity, a requisite event for muscle repair [58]. The cytokine receptor is expressed in mouse SCs that have exited quiescence and are considered capable of mitotic activity [59]. Stimulation of SCs with G-CSF, the ligand for CSF3R, results in a greater proportion of self-renewing SC, ensuring replenishment of the stem cell population [60]. Our results extend those found in mice to the horse and provide indirect evidence that MSM advances the muscle repair process following exercise.

## 5. Conclusions

In summary, the skeletal muscle transcript profiling of samples isolated prior to exercise reveals no changes in gene expression, arguing that MSM did not affect the expression of the genes broadly classified as antioxidants and anti-inflammatories. However, our results demonstrate that MSM does affect the response to exercise, with a greater expression of genes driving neutrophil invasion and macrophage activity, suggesting that it may accelerate the initial stages of muscle repair. By examining the tissue at later time points (6, 12, and 24 h), a more comprehensive overview of the inflammatory response to exercise could be developed to address the role of MSM as a priming agent that facilitates the rapid repair of the damaged tissue.

## Figures and Tables

**Table 1:** Nutrient composition of feedstuffs on a dry matter basis.

Component ^1^	Concentrate ^2^	Hay ^3^
DM, %	89.4	92.3
CP, %	13.2	9.7
ADF, %	24.4	40.5
NDF, %	33.5	63.5
Starch, %	10.1	0.3
Fat, %	14.3	3.3
Ash, %	9.8	5.8
Calcium, ppm	14,100	4160
Chloride, ppm	5590	Bdl ^4^
Copper, ppm	92	5
Iron, ppm	1420	134
Magnesium, ppm	4100	2540
Manganese, ppm	323	120
Phosphorus, ppm	5300	2730
Potassium, ppm	14,800	20,600
Sodium, ppm	3100	281
Sulfur, ppm	4000	n/a
Zinc, ppm	377	18
Calculated DE, Mcal/kg DM	3.27	2.13

^1^ DM, dry matter; CP, crude protein; ADF, acid detergent fiber; NDF, neutral detergent fiber; DE, digestible energy. ^2^ Ultium Competition, Purina Mills, Arden Hill, MO, USA. ^3^ Orchard grass. ^4^ n/a: below detection limits.

**Table 2:** Genes differentially expressed by 2-fold or more in response to exercise. Downregulated genes are shown in **BOLD**.

Gene ID	Gene Name	Log Fold Change
NR4A3	nuclear receptor subfamily 4 group A member 3	4.886
PDK4	pyruvate dehydrogenase kinase 4	4.734
NR4A2	nuclear receptor subfamily 4 group A member 2	4.403
DMP1	dentin matrix acidic phosphoprotein 1	4.066
HSPA6	heat shock protein family A (Hsp70) member 6	4.021
THBS1	thrombospondin 1	3.399
ATF3	activating transcription factor 3	3.341
CISH	cytokine inducible SH2 containing protein	3.340
HSPH1	heat shock protein family H (Hsp110) member 1	3.249
SIK1	salt inducible kinase 1	3.234
CHAC1	ChaC glutathione specific gamma-glutamylcyclotransferase 1	3.089
MFSD2A	major facilitator superfamily domain containing 2A	3.025
GADD45G	growth arrest and DNA damage-inducible gamma	2.894
FOS	Fos proto-oncogene, AP-1 transcription factor subunit	2.739
MAFF	MAF bZIP transcription factor F	2.536
SERPINE1	serpin family E member 1	2.532
APOLD1	apolipoprotein L domain containing 1	2.486
TCIM	transcriptional and immune response regulator	2.461
CREB5	cAMP responsive element binding protein 5	2.420
CEBPD	CCAAT enhancer binding protein delta	2.405
**APLNR**	**apelin receptor**	**−2.377**
CNN1	calponin 1	2.373
ANGPTL4	angiopoietin-like 4	2.307
PER1	period circadian regulator 1	2.283
FOSL2	FOS like 2, AP-1 transcription factor subunit	2.252
SGK1	serum/glucocorticoid-regulated kinase 1	2.194
CIART	circadian-associated repressor of transcription	2.171
ITGA5	integrin subunit alpha 5	2.161
IRS2	insulin receptor substrate 2	2.144
ARRDC4	arrestin domain containing 4	2.115
CDKN1A	cyclin-dependent kinase inhibitor 1A	2.103
CSRNP1	cysteine- and serine-rich nuclear protein 1	2.053
DNAJB1	DnaJ heat shock protein family (Hsp40) member B1	2.047
SDC4	syndecan 4	2.035
OTUD1	OTU deubiquitinase 1	2.031

**Table 3:** Exercise response genes differentially expressed in MSM-supplemented adult thoroughbred geldings over-represented in immune system pathways—hierarchal organization of selected major subclasses denoted by indentation.

Pathway		Number of Mapped Genes	Representative Genes
Adaptive immune system		30	ASB18, CALM2, CALR, CAP1, CAPZA2, CTSB, DCTN6, ERAP1, FBXO9, ITGB2, KLRG1, MYD88, ORAI2, PJA1, PPP2R5B PTPN6, RCHY1, RNF182, RNF41, RNF6, S100A9, SEC61A1, SLA, TLR1, TREML2, TRIM11, TUBB4B, TYROBP, UBA3
Innate immune system		68	ABL1, ACTR10, ADGRE5, AGL, ALOX5, ARPC1B, ARPC5, BIN2, BORC2, C4BPA, C5AR1, CALM2, CAP1, CAPZA2, CD55, CDA, CFI, CNN2, COTL1, CTSB, FCAR, FCER1G, FGR, FTL, GLA, GMFG, GRN, ITGAM, ITGB2, MAP2K4, MCEMP1, MME, MYD88, MYH9, MYO9B, NCKAP1L, NFAM1, NLRC4, PLAUR, PLEKHO2, PLIN3, POLR1C, PSEN1, PTPN6, RAB31, RAP2C, RPS6KA1, S100A9, S100P, SERPINB1, SIKE1, SIRPA, SLC11A1, STK10, TBK1, TLR1, TLR5, TLR8, TNFAIP6, TRPM2, TUBB4B, TXK, TYROBP, UBA3, VNN2
	Toll-like receptor cascades	15	IRF5, ITGAM, TLR8, CTSB, ITGB2, MAP2K4, TBK1, TLR5, CAP1, BIRC2, MYD88, TLR1, PLIN3, S100A9, RPS6KA1
Cytokine signaling in the immune system		38	ALOX5, ASB18, BCL6, BIRC2, CAP1, CASP3, CNN2, CSF1, CSF2RB, CSF3R, EIF4E3, FLNA, FSCN1, HCK, HNRNPF, IL10RA, IL10RB, IL1RN, IL6R, IRF4, IRF5, ITGAM, ITGB2, MAP2K4, MMP1, MYD88, NUP98, NUPL2, PTPN1, PTPN6, RPS6KA1, SLA, SOCS6, STAT3, TBL1, NFSF12, TUBB4B, UBA3
	Interferon signaling	11	FLNA, NUP98, IRF5, EIF4E3, IRF4, PTPN1, NUPL2, TUBB4B, PTPN6, STAT3, ASB18,
	Interleukin signaling	24	IL6R, IL10RA, MMP1, IL10RB, PTPN6, MAP2K4, TBK1, HNRNPF, CNN2, RPS6KA1, CASP3, IRF4, HCK, CSF3R, FSCN1, CSF1, ITGB2, ITGAM, MYD88, BCL6, ALOX5, CSF2RB, STAT3, IL1RN

**Table 4:** Exercise response genes differentially expressed in CON-supplemented adult thoroughbred geldings over-represented in metabolism and signal transduction pathways.

Pathway	Number of Mapped Genes	Representative Genes
Metabolism	30	ACSL5, ADA, AZIN1, G3GALT2, BDH2, CSGALNACT1, GDPD3, GGPS1, GNG2, HAL, HAD17B11, LDLR, MED13L, MFSD2B, NAGLU, NUDT4, NUP153, PARP8, PIK3CA, PIP5K1C, PLEKHA2, PPP2R5D, SC5D, SEC24A, SEPHS2, SIRT4, SLC24A13, SLC25A22, TST, UCK2
Signal transduction	42	ACTR3, ADRB2, AGO3, BMP2, CCL16, CDK5R1, CXCL1, DGKE, DLL4, DUSP7, F11R, F2R, FAM91A1, FGD5, GNG2, HDAC7, IL6ST, KPNA2, LEMD3, PDE4D, PDGFRB, PENK, PIK3CA, PIP5K1C, PKN3, PLXNB1, PLXND1, POGLUT1, PPP2R5D, PYGO2, RET, RRH, SLK, SMAD9, SNAI2, SOX4, SOX9, TNRC6B, USF1, WDR6

## Data Availability

The data presented in this study are available as BioProject PRJNA1186588 through the National Center for Biotechnology Information.
